# Driven Engulfment of Janus Particles by Giant Vesicles in and out of Thermal Equilibrium

**DOI:** 10.3390/nano12091434

**Published:** 2022-04-22

**Authors:** Vaibhav Sharma, Carlos M. Marques, Antonio Stocco

**Affiliations:** Institute Charles Sadron, CNRS UPR22, University of Strasbourg, 23 Rue du Loess, 67034 Strasbourg, France; vaibhav.sharma@etu.unistra.fr (V.S.); marques@unistra.fr (C.M.M.)

**Keywords:** Janus particles, lipids, vesicles, engulfment, adhesion, self-propulsion, driven interaction

## Abstract

The interaction between Janus colloids and giant lipid vesicles was experimentally investigated to elucidate the dynamics and mechanisms related to microparticle engulfment by lipid vesicles. Janus (Pt–SiO_2_ and Pt–MF, where MF is melamine formaldehyde) colloids do not spontaneously adhere to POPC or DOPC bilayers, but by applying external forces via centrifugation we were able to force the contact between the particles and the membranes, which may result in a partial engulfment state of the particle. Surface properties of the Janus colloids play a crucial role in the driven particle engulfment by vesicles. Engulfment of the silica and platinum regions of the Janus particles can be observed, whereas the polymer (MF) region does not show any affinity towards the lipid bilayer. By using fluorescence microscopy, we were able to monitor the particle orientation and measure the rotational dynamics of a single Janus particle engulfed by a vesicle. By adding hydrogen peroxide to the solution, particle self-propulsion was used to perform an active transport of a giant vesicle by a single active particle. Finally, we observe that partially engulfed particles experience a membrane curvature-induced force, which pushes the colloids towards the bottom where the membrane curvature is the lowest.

## 1. Introduction

Micro- and nano-particles interacting with cell membranes made of lipid bilayers may induce membrane poration, rupture, particle engulfment or simply repulsion [1,2,3,4]. Hence, understanding and controlling particle-lipid membrane interaction is crucial for drug delivery, microbial infection, toxicity from microplastics and in many other fields [1]. Biological cells can use biological or chemical functions to interact with microparticles in order to perform tasks as phagocytosis, endocytosis or exocytosis [1,5]. In particle endocytosis, the penetration of a microparticle inside the cell occurs upon particle engulfment by the lipid membrane. Once penetrated, the particle is fully wrapped by a lipid membrane, which avoids direct contact between the particle and the cell interior. Microparticle engulfment can also occur in biomimetic systems such as giant unilamellar vesicles (GUVs) [6] in the absence of any active biological mechanisms. Particle adhesion on the membrane can in fact overcome the bending and stretching costs of the membrane; and adhesive particles can be partially or completely engulfed by GUVs in a spontaneous manner, depending on the adhesion strength [3,7]. Although moderately to strongly adhesive particles have been well studied, much less attention has been paid to the spontaneous engulfment of microparticles by GUVs in the case of weak adhesion [8,9]. Only recently, Spanke et al. investigated the interaction of POPC GUVs with polystyrene (PS) microparticles and observed that particle engulfment by a GUV is only possible for very low membrane tensions (<10^−8^ N m^−1^) and in the presence of attractive depletion interaction, which can be tuned by adding polymers in the aqueous medium [10]. For iron oxide–PS Janus particles, Ewins et al. have very recently shown that positively charged GUVs (DOPC containing a small percentage of DOTAP) are able to engulf negatively charged PS particles and PS-based Janus particles [11]. Membrane wrapping occurs for low membrane tensions, and partial engulfment of Janus particles and complete engulfment of bare PS particles can be observed.

In the last years, self-propelled Pt-coated Janus particles (moving with persistent speeds of few µm/s) have been also investigated, either when they were encapsulated inside GUVs or when they moved close to GUVs [12,13]. Fascinating dynamics such as vesicle shape deformation, active membrane fluctuations and particle orbital motion around GUVs have been reported in these systems.

In this article, we report a systematic experimental investigation on the interaction between Janus colloids and phospholipid GUVs in order to elucidate whether particle engulfment can be driven by external forces alone, without introducing significant chemical or physical modifications in the system. We consider two lipid systems and particles made of silica, platinum and melamine resin. We focus our attention on the non-specific adhesion of the Janus particle regions on the GUV membrane, which can be trigged by the application of an external force. Finally, after having achieved control of the partial engulfment state of a Janus particle, we explore the ability of the self-propelled particle to drive GUV transportation in this partially engulfed geometry.

## 2. Materials and Methods

### 2.1. Colloids and Vesicles

We used bare silica microspheres of radius *R*_P_ = 1.96 ± 0.05 µm, and fluorescent melamine formaldehyde resin (MF) colloids *R*_P_ = 1.29 ± 0.06 µm (microParticle GmbH, Berlin, Germany). Janus colloids were fabricated following the method proposed by Love et al. [14]. First, a monolayer of either silica or MF beads was formed on a cleaned silicon wafer by drop casting (a particle solution of 2% concentration by volume). After drying, a thin layer of platinum was deposited on the colloidal monolayer using a metal sputtering contraption (Auto 306 Evaporator, BOC Edwards, West Sussex, UK). Metal sputtering yields particles that are partially coated by platinum, showing a thickness = 6 ± 1 nm as measured by light reflectivity (Multiskop, Optrel, Berlin, Germany). GUVs are prepared using PVA (polyvinyl alcohol) gel assisted formation [15]. In this method, PVA gel is prepared by dissolving dry PVA in PBS (phosphate-buffered saline, 10 g L^−1^) solution at 5% concentration. The prepared PVA gel is then spread inside a homemade PTFE (polytetrafluoroethylene) plate chamber and dried for 30 min at 80 °C. 10 mL of a 99:1 (molar) mixture of either POPC-NBD (1-palmitoyl-2-oleoylphosphatidylcholine fluorescently labelled with nitrobenzoxadiazole) or DOPC-NBD (1,2-dioleoyl-sn-glycero-3-phosphocholine fluorescently labelled with NBD) lipids in chloroform (1 g L^−1^) are spread on the PVA gel and vacuum dried for 15 min. The lipids are then hydrated with 200 mL of sucrose (0.15 M) and allowed to grow for at least 2–3 h. The vesicle suspension after growing is collected and sedimented in 1 mL of glucose (0.15 M) solution. The very slight density mismatch Δρ between the solutions inside the vesicle and outside in the aqueous medium allows the vesicles to sediment at the bottom of the collection tube. The average radius for GUVs obtained from PVA gel assisted formation method was measured to be *R*_GUV_ = 11 ± 5 µm. 

### 2.2. Centrifugation

**Figure 1:**
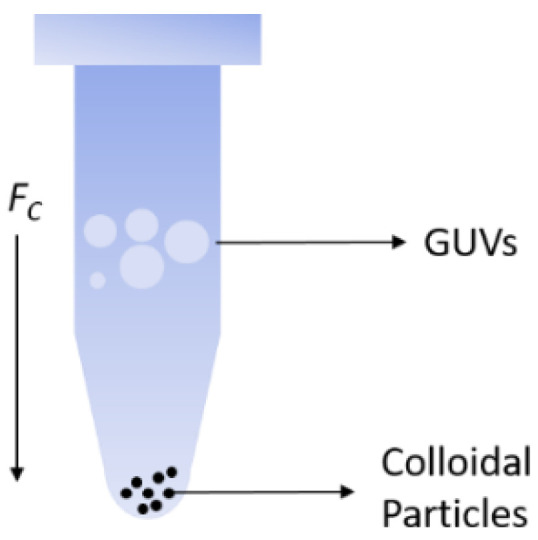
Schematic of force driven interaction between Janus colloids and GUVs by centrifugation.

Janus colloids were released from the wafer using 10 mL of glucose solution (0.1 M) along with simple agitation using a micro pipet tip. The glucose–colloidal suspension was then added to a 1 mL centrifugation tube with 120 µL of glucose solution. The particles were then sedimented to the bottom using centrifugation using high centrifugation speeds. The particles, being heavier, did not require a long time to sediment. After the colloids were sedimented to the bottom, 1 µL of GUV solution was added to the centrifugation tube (Figure 1), which was then centrifuged and brought into contact with the Janus colloids at the bottom of the tube with different centrifugation speeds (*RPM*) for 4 min. This study primarily focuses on the relative centrifugal force $RCF=1.12\;r\;{\left(RPM/1000\right)}^{2}$ (where *r* = 95 mm is the rotor radius) in the range RCF = 106–1700 *g*, where *g* is the gravity acceleration constant *g* = 9.8 m s^−2^.

**Figure 2:**
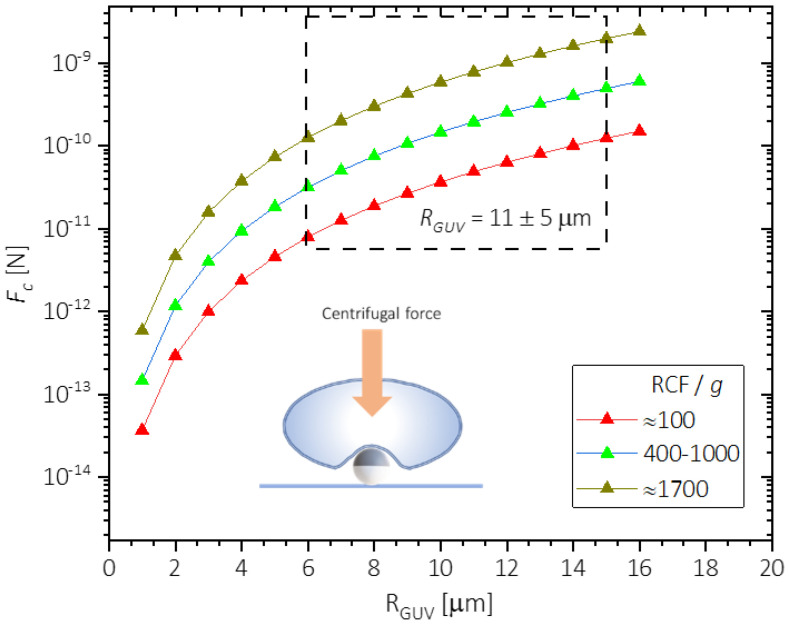
Calculated centrifugal force (*F*_c_) as a function of the vesicles radius (*R*_GUV_) for different relative centrifugation force, *RCF*.

As the colloids were already sedimented to the bottom before adding the GUVs, the centrifugation force $F_{C}=4/3\pi R_{GUV}^{3}\;\Delta \rho \;RCF\;$ only acted on the vesicles. Calculated $F_{C}$ as a function of vesicle radius *R*_GUV_ is shown in Figure 2 for three different levels of *RCF* corresponding to: low forces *RCF*/*g* ≈ 100, moderate forces 400 ≤ *RCF*/*g* ≤ 1000 and high forces *RCF*/*g* ≈ 1700. A window of the force experienced by the GUVs is shown in Figure 2, considering the GUV radius distribution (*R*_GUV_ = 11 ± 5 µm) by gel formation method. 

### 2.3. Microscopy and Tracking

Both bright-field and fluorescent microscopies were used to observe the Janus colloids and GUVs in aqueous solutions. The Janus–GUV dispersion after centrifugation was transferred to the sample cell (≈130 µL). The optical setup consisted of Nikon (Tokyo, Japan) Eclipse TE2000 microscope (×60 objective) equipped with a CMOS camera (Orca Flash 4.0, Hamamatsu, Hamamatsu, Japan). Videos were recorded in a 10–1000 frame per second (fps) range. Tracking the particle centre of mass (COM) was achieved using the open-source software Blender v 2.8 (Blender Foundation, Amsterdam, The Netherlands). Tracking the particle orientation was restricted to MF–Pt colloids. Fluorescence microscopy was used to unambiguously determine the particle out-of-plane orientation angle. The videos were recorded at high frame rates (≈1000 fps) and analysed by threshold technique using ImageJ (NIH, Bethesda, MD, USA). The analysis yields a brighter area, corresponding to the MF part of the Janus colloid, observed by florescence microscopy in reflection. This area is a function of the out-of-plane particle orientation angle, which was used to calculate the mean squared angular displacement as a function of the lag time ∆*t* [16].

## 3. Results and Discussion

### 3.1. Interaction between Janus Particles and GUVs Driven by Centrifugation

**Figure 3:**
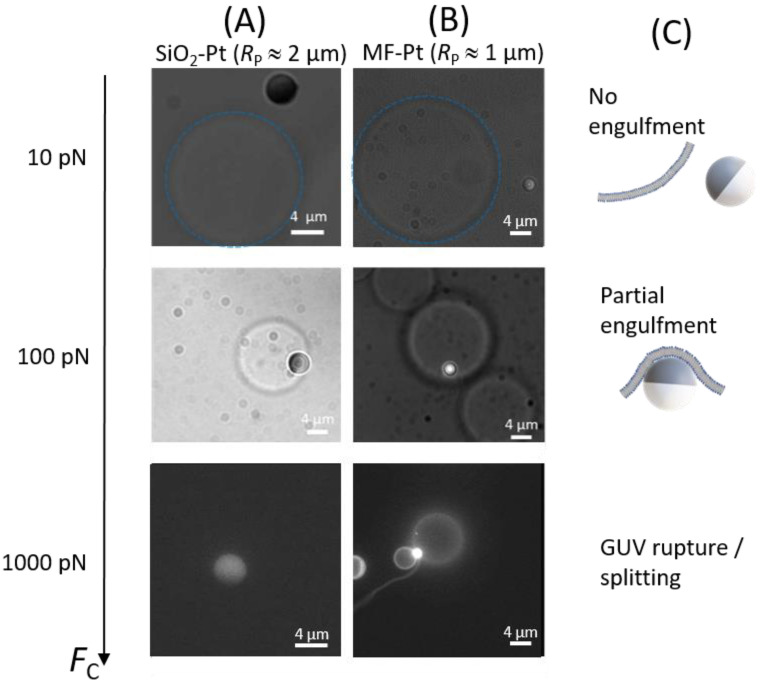
Bright field and fluorescence microscopy images of: (**A**) SiO_2_–Pt Janus colloids (*R*_P_ ≈ 2 µm) and (**B**) MF–Pt Janus colloids (*R*_P_ ≈ 1 µm) interacting with POPC GUVs at given centrifugal forces *F*_C_. Images are taken from below. Dashed blue circles are guides to identify the vesicles. (**C**) Sketches of a Janus colloid non-interacting with a membrane at *F*_C_ ≈ 10 pN, and a Janus colloid partially engulfed by a GUV membrane at *F*_C_ ≈ 100 pN. For *F*_C_ ≈ 1000 pN, the number and the size of GUVs dramatically decreased due to GUV rupture and splitting.

In thermal equilibrium, both SiO_2_–Pt and MF–Pt Janus colloids do not show any tendency to adhere to GUVs made of POPC or DOPC. In order to drive the particle–GUV interaction, we investigated the effect of the centrifugation force on the particle engulfment by GUV [17]. As in thermal equilibrium, also at low centrifugal forces $F_{C}$ ≈ 10 pN, no particle engulfment by GUVs was observed for SiO_2_–Pt or MF–Pt Janus colloids. After centrifugation, the Janus colloids behaved as before, diffusing close to GUVs but without showing any adhesion or engulfment by the GUVs, see Figure 3 (top images).

By increasing $F_{C}$ by one order of magnitude, $F_{C}$ ≈ 100 pN, we observed engulfment of Janus colloids by GUVs. For SiO_2_–Pt Janus colloids, the Pt region seems to adhere to the GUV membrane, as shown in the bright field microscopy image of Figure 3 (A-column, center) where the darker part of the colloid correspond to Pt. Fluorescence microscopy shows that MF–Pt Janus colloids are located under the GUV with the MF region facing the bottom of the cell and the Pt region engulfed by the GUV (Figure 3, B-column, center). As will be described in Section 3.2, partially engulfed MF–Pt Janus colloids hardly change their out-of-plane orientation. They appear as bright circles similar to bare particles. Note that the Pt coating of the particle is thick enough to shield the fluorescence and an isolated Janus MF–Pt colloid far from a GUV appears as a moon in its principal lunar phases (see Section 3.2). The partial engulfment of particles was mainly observed for MF–Pt Janus colloids (*R*_P_ ≈ 1 µm), while for the larger SiO_2_–Pt colloids (*R*_P_ ≈ 2 µm) partial engulfment remained a fairly rare observation. In the following, we will first elucidate whether partial engulfment driven by $F_{C}$ results from the adhesion of only the Pt region for MF–Pt particles. This adhesion is due to non-specific interactions as in the physics of wetting. Note that both the vesicle and the particle possess slightly negative surface potentials [18,19,20]. Hence, membrane engulfment or adhesion is not driven by charge effects [11]. For SiO_2_–Pt colloids, the rare partial engulfment observed will be discussed as a consequence of a complete membrane adhesion on the colloid’s surface, which may result in a full coating of the particle by the lipids and lead to membrane rupture in the GUVs.

**Figure 4:**
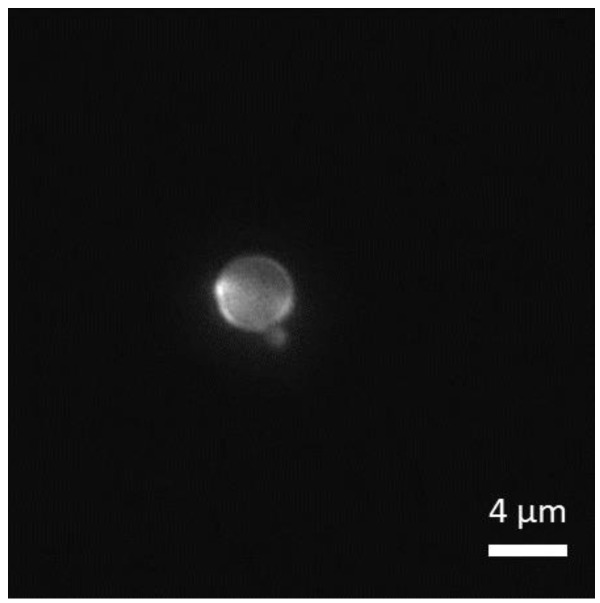
Fluorescence image of a bare (non-fluorescent) silica colloid after GUVs centrifugation at *F*_C_ ≈ 1000 pN. Fluorescent regions on the particles are due to the adsorption of lipids onto silica.

At higher forces, $F_{C}$ ≈ 1000 pN, Janus particles were found to either split a GUV into two or three vesicles, or to destroy the GUVs, see Figure 3 (bottom image, B-column). For both Janus colloids, after centrifugation the number and the size of GUVs severely decreased, pointing to vesicle rupture due to an overly strong driven interaction between the membranes and Janus colloids. Indeed, we observed that non-fluorescent SiO_2_–Pt Janus colloids became fluorescent due to (the fluorescently labelled) lipid adsorption on the particle (Figure 3, A-column, bottom). In many cases, MF–Pt Janus colloids were found between small GUVs or tubes, which could represent the result of a GUV splitting or rupture process (Figure 3, B-column, bottom). Table 1 and Table 2 summarize the results of all our observations (more than 200). For MF–Pt Janus colloids and $F_{C}$ ≈ 100 pN, many particle partial engulfment states were observed in both DOPC and POPC GUVs. On the contrary, SiO_2_–Pt Janus colloids in many cases either show no interaction with GUVs or they are able to destroy them because of an activated adhesion on both silica and platinum (see Figure 3, A-column, bottom and Figure 4). For DOPC GUVs, vesicle rupture and adsorption of fluorescently labelled lipids on SiO_2_–Pt Janus colloids was observed even at lower forces. Note that DOPC is slightly more fluid than POPC (melting temperature T_m_ (DOPC) = −17 °C and T_m_ (POPC) = −2 °C), and leaflets show a slightly spontaneous negative curvature *C*_l_ (DOPC) = −70 µm^−1^, whereas *C*_l_ (POPC) ≈ 0 [21]. Although the bilayer spontaneous curvature is zero, adhesion of the particles to one of the leaflets is likely to locally change the conformation of the lipids in that leaflet, therefore creating an asymmetric state where the leaflet spontaneous curvature plays a role. These lipid properties could be related to the higher tendency of DOPC to adhere to the colloids with respect to POPC. Note that for bare polystyrene particles, engulfment by GUV was observed in DOPC vesicles under the action of centrifugal forces [17], whereas no particle engulfment was observed for POPC vesicles under the action of optical forces (≈1 pN) [10].

### 3.2. Partial Engulfment of Janus Particles by GUVs

In order to understand if GUV rupture events are related to an activated adsorption (by *F*_C_) of both silica and platinum on the GUV membranes, and if partial engulfment of Janus particles can be achieved by colloids showing one adhesive face (Pt) and one non-adhesive face (MF), we also investigated bare (non-Janus) particles interacting with GUVs. 

We performed the same GUV centrifugation experiments as before, using bare (non-fluorescent) silica and non-fluorescent MF colloids (microParticles GmbH).

We observed that bare silica colloids (*R*_P_ ≈ 1 and 2 µm), as with SiO_2_–Pt Janus colloids, become fluorescent after GUV centrifugation at $F_{C}$ ≈ 1000 pN, see Figure 4. As before, the number and the size of GUVs severely decreased after centrifugation. On the contrary, (non-fluorescent) bare MF colloids do not become fluorescent even when GUV rupture was observed. These results confirm that adhesion between silica and lipid bilayers can be driven by $F_{C}$, whereas the adhesion of MF cannot be activated by $F_{C}$.

**Figure 5:**
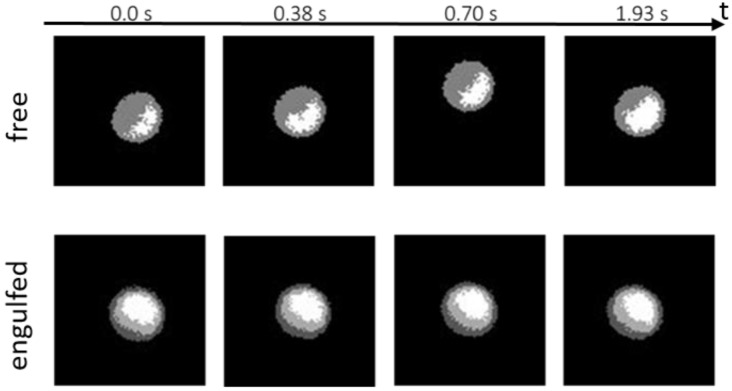
Fluorescence microscopy images taken at different experimental time (0, 0.38, 0.7 and 1.93 s) of an isolated MF–Pt Janus colloid (*R*_P_ ≈ 1 µm) far away from any GUVs (**top panel**) and a Janus colloid partially engulfed by a GUV (**bottom panel**).

Now we focus our attention on the rotational dynamics of single MF–Pt colloids, which are partially engulfed by GUVs. In Figure 5, some fluorescence microscopy images of partially engulfed Janus colloids are compared to images of isolated MF–Pt Janus colloids far from GUVs. Isolated Janus colloids are free to rotate and they are able to change their in-plane and out-of-plane orientation in time. Partially engulfed Janus colloids instead seem to not change their orientation significantly.

**Figure 6:**
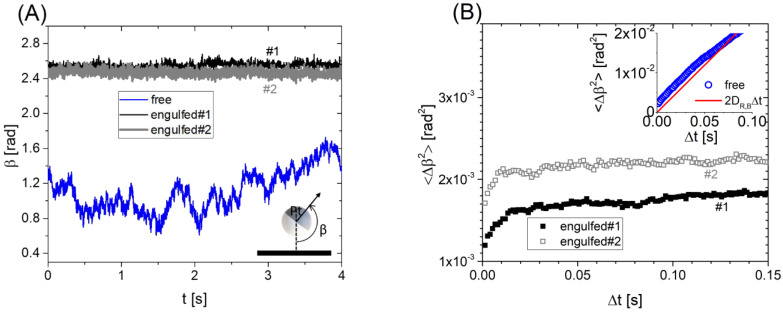
(**A**) Out-of-plane particle orientation angle *β* as a function of time *t* for a free isolated Janus colloid and two partially engulfed Janus colloids by GUVs. The sketch in the inset shows the definition of *β*. (**B**) Mean squared angular displacements <∆*β*^2^> as a function of the lag time ∆*t* corresponding to the data shown in (**A**). In the inset, <∆*β*^2^> data for a “free” colloid are compared with the Brownian rotational dynamics prediction: <∆*β*^2^> = 2*D*_R,B_∆*t*.

As explained in Section 2.3, the bright area (*A_MF_*) observed under fluorescence microscopy corresponds to the MF region of the Janus colloid, whereas the missing area within the radius of the circle corresponds to the Pt region. From these areas, we evaluated the out-of-plane orientation angle *β*: $A_{MF}=1/2\pi \left(1-\cos\beta \right)R_{P}^{2}$, see Figure 6A, and plot it as a function of time [16]. Clearly, the isolated Janus colloid is “free” to rotate and *β* varies between 0.6 to 1.7 rad (34° to 97°) in 4 s. For engulfed Janus colloids, *β* varies only in a narrow range: ∆*β* ≈ 0.1 rad with a typical variance of 10^−3^ rad^2^. In Figure 6B, mean squared angular displacements <∆*β*^2^> as a function of the lag time are shown for the “free” and “engulfed” Janus colloids. For the isolated Janus colloid, <∆*β*^2^> changes linearly with the lag time and can be compared to the Brownian rotational dynamics of a spherical particle in the bulk, <∆*β*^2^> = 2 *D*_R,B_∆*t* where $D_{R,B}=\frac{k_{B}T}{8\pi \eta R_{P}^{3}}$ is the rotational diffusion $k_{B}T$ is the thermal agitation energy and η = 1.02 is the fluid viscosity [13]. Note that a correction to the bulk rotational diffusion should be considered due to the presence of a wall on the bottom [22]. However, this correction is small, considering typical gap distances between the particle and the bottom wall [23]. Janus particles partially engulfed by GUVs show <∆*β*^2^> increasing only at very short lag times (<0.01 s), and reach some plateau values that confirm a confinement in the particle orientation dynamics.

This confinement could be due to the preferential affinity of only one face of the Janus colloids towards the GUV membrane and can be rationalized in terms of the adhesion energy of the system. 

As sketched in Figure 3C (center), we can start by assuming an initial particle orientation with the entire Pt region oriented towards the GUV membrane (*m*) and the entire MF region oriented towards the aqueous solution (*aq*) on the bottom. In order to change orientation by ∆β* an area fraction of the Pt region should “dewet” from the GUV membrane and become wetted by the aqueous solution. In turns, an area fraction of the MF region should do the opposite. The area of the particle involved in this orientation change is simply $f=2R_{P}^{2}\Delta \beta^{*}$ and the change in free energy can be written as: $\Delta E\left(\Delta \beta^{*}\right)=f\Delta W,$ where ΔW is the change in membrane–particle adhesion between a bare MF region and a Pt covered area. By thermal equipartition one has for equilibrium fluctuations: $<\Delta E\left(\Delta \beta^{*}\right)>\;=1/2k_{B}T$, where $\Delta \beta^{*}$ correponds to the typical particle orientation variations shown in Figure 6. Hence, we calculate ΔW= 1–5 × 10^−8^ N m^−1^, consistent as it should be with the order of magnitude of membrane tensions measured for floppy GUVs [10]. Indeed, finite contact angles between an adhered membrane and a surface require the contact adhesion energy between the membrane and surface to be of the same order of magnitude [6]. Note that we assumed a negligible rotational motion of the vesicle given that: (i) the bulk rotational diffusion coefficient *D*_R,B_ of the GUV (*R*_GUV_ = 11 ± 5 µm) is typically 1000 times slower than the particle one; (ii) the slowing down of the rotation due to the bottom wall impacts the GUV more than the particle, given the non-spherical vesicle shape close to the wall due to gravity; and (iii) the confined particle rotational diffusion measured at short lag times (Figure 6B) is on average only 4 times slower than the bulk one.

Finally, these results confirm that different engulfment states of Janus colloids by GUVs can be achieved by controlling the adhesion properties of each face of the Janus particles. It is important to recall that these adhesion energies and the corresponding tensions are weak and a nanometric water gap always exists between the solid particle and the lipid membrane in the absence of strong membrane binding [24,25].

### 3.3. Self-Propelled Janus Particles Partially Engulfed by GUVs

Janus colloids are able to self-propel, moving with persistent speed *V* of few µm/s, due to the reaction of hydrogen peroxide on the Pt region of the colloid [13,26,27,28]. In these out-of-equilibrium conditions, particle self-propelled motion usually occurs close to the bottom solid boundary, which polarizes the out-of-plane particle orientation around *β* = π/2, with the Janus particle boundary oriented perpendicular to the bottom wall [29].

**Figure 7:**
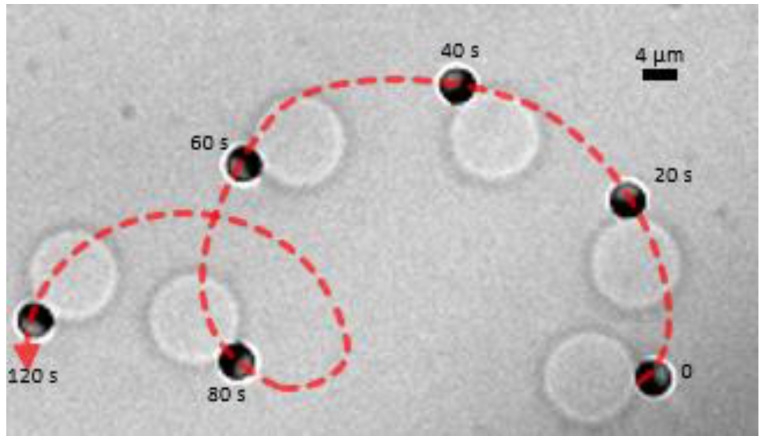
Time-lapse bright field optical microscopy images of the active transport of a ≈12 µm giant POPC vesicle by a Pt–SiO_2_ Janus microparticle (Pt is the darkest region in the image) self-propelling in a 2% H_2_O_2_ solution. The experimental time *t*(s) is shown close to the microparticle at each snapshot. Dashed red line shows the smoothed particle trajectory.

In our experimental system, we added a small volume of a concentrated H_2_O_2_ solution to reach a 2% concentration in the aqueous solution to trigger the self-propulsion of Janus colloids that were already partially engulfed by the GUVs [13]. Note that no significant changes of the GUV properties were observed due to a 2% H_2_O_2_ in the solution [13]. Figure 7 shows the active transport of a GUV by a SiO_2_–Pt Janus colloid. A portion of the GUV membrane adheres to a small region of Pt face of the Janus particle, which is able to drag the vesicle over very long distances at speed *V* ≈ 1 µm/s, which is smaller but comparable to the speed *V* ≈ 1–4 µm/s measured for isolated particles [13]. Note that in Figure 7 the particle out-of-plane orientation is *β* ≈ π/2. As previously noted in Table 2, these observations were rare but demonstrate a strategy to perform active GUV transport by a self-propelled colloid, when the partial engulfment by the GUV occurs in a small particle region.

Adding hydrogen peroxide to a dispersion containing MF–Pt Janus colloids partially engulfed by GUVs, in general, does not result in the observation of active GUV transport. As discussed in the previous section, before adding H_2_O_2_ the entire Pt region of the MF–Pt particle may adhere to the GUV membrane and partially engulfed Janus colloids show out-of-plane orientation *β* close to π (i.e., 4/5π, see Figure 6A). The same orientation is found also after adding hydrogen peroxide at long observation times, despite the fact that for self-propelled Janus colloids *β* ≈ π/2 is expected, see Figure 7.

**Figure 8:**
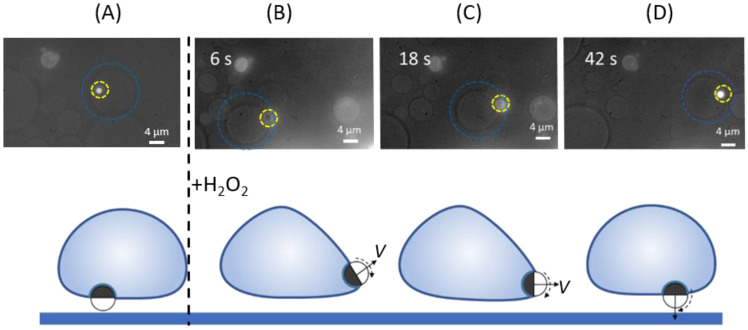
(**Top panel**) Fluorescence microscopy images of a MF–Pt Janus colloid partially engulfed by a GUV before (**A**) and after (**B**–**D**) adding hydrogen peroxide in the aqueous solution. Dashed circles are guides to identify the particle (yellow) and the GUV (blue). Images are taken from below. (**Bottom panel**) Sketches of the scenarios describing the colloid–GUV configurations observed in the experiments above.

In Figure 8, we were able to monitor the changes induced by the addition of hydrogen peroxide, and discuss a transient active transport of a GUV by a partially engulfed MF–Pt Janus. We focused our attention on a partially engulfed MF–Pt Janus colloid in thermal equilibrium (before adding H_2_O_2_): the particle appears as a bright circle in fluorescent microscopy and the out-of-plane orientation *β* is close to π, see Figure 8A. Hence, we were able to monitor the particle–GUV system a short time after adding H_2_O_2_. Figure 8B shows that the Janus particle moves toward the opposite periphery of the GUV and significantly changes its orientation *β*, losing its brightness. For approximately 30 s the Janus particle is able to self-propel and drag the GUV, see Figure 8B,C. In this time interval, the Janus particle’s orientation *β* changes again, with a bright area appearing on front of the Janus particle. In the last stages, *β* returns to its initial value close to π, quenching the active motion of the particle–GUV system, see Figure 8D.

We sketched this transient phenomenon on the bottom panel of Figure 8. In the initial particle configuration, before adding H_2_O_2_, the Janus colloid is in a central region on the bottom of a GUV. A competition between the initial particle orientation (*β* = π) and the self-propelled particle orientation (*β* = π/2) occurs in the transient regime. A self-propelled colloid is able to drag the GUV because its orientation changes from *β* = π, leading to a non-zero velocity component parallel to the bottom substrate (sketches in Figure 8B,C). Since we observed a growing bright area on the front of the particle, the Janus colloid starts with the Pt region oriented towards the bottom; it then rotates clockwise until the entire MF region is oriented again downwards. These transient dynamics can be explained as the result of a competition between a rapid particle orientation change due to the addition of hydrogen peroxide (particle self-propulsion), and a membrane curvature-induced force.

Recently, a membrane curvature-induced force was predicted by Agudo–Canalejo and Lipowsky [30]. The partial engulfment of a colloid by a GUV leads to a variation of the energy landscape of the GUV since the membrane curvature has to change close to the particle region. For GUVs showing asymmetrical shapes (i.e., non-spherical), gradients of the membrane curvature exist even before the particle engulfment. Hence, as a consequence of both the particle engulfment and the original GUV membrane curvature gradient, the partially engulfed particle experiences a curvature-induced force towards the region of the GUV with the lowest membrane curvature, which represents the minimum of the global free energy in the system.

In our experiments, the GUV possessed a density that is higher than the aqueous solution, which lead to a flattening of the membrane area on the bottom. This explains why MF-Janus colloids were always in a central region on the bottom of a GUV, where the membrane curvature is the lowest. A quick perturbation of the particle orientation, which tends to *β* = π/2 in the presence of H_2_O_2_, was then quenched by the GUV membrane, which tends to reduce its deformation by pulling the particle from the periphery to a central region. In Figure 8D, even if the particle possesses a persistent speed *V*, the velocity component parallel to the bottom wall is negligible, given that the Pt region is oriented opposite to the bottom wall (*β* ≈ π). Hence, the active motion is suppressed because the self-propulsion velocity is oriented towards the bottom wall, see the sketch in Figure 8D.

These results indicate that the effect of a membrane curvature-induced force should be taken into account in order to achieve active transportation of a GUV by a partially engulfed self-propelled particle. As shown in Figure 7, this force can be minimized by decreasing the adhesion area between the particle and the GUV.

## 4. Conclusions

We have demonstrated that engulfment of Janus particles by GUVs can be driven by an external centrifugal force $F_{C}$ and in the absence of strong chemical or physical modifications in the system: strong membrane binding, electrostatic attraction, depletion interaction, and very low membrane tension [9,10,11]. This force $F_{C}$ is required to overcome an energy barrier for adhesion and engulfment, which may result from electrostatics, repulsion due to membrane fluctuations, or membrane stretching and bending [7,10,31,32]. Given the absence of specific chemical interactions, the adhesion in our systems is weak and in the range of 1 to 5 × 10^−8^ N m^−1^. Adhesion of Janus particles show similarities with adhesion observed in supported bilayers or GUVs on solid substrates, showing that lipid bilayers have affinities for silica and platinum but not for most polymeric surfaces [24,33,34]. Partial engulfment by GUVs can be achieved for $F_{C}$ ≈ 100 pN and for Janus particles showing only one adhesive particle region. We have also observed that a self-propelled Janus particle, which adheres to the membrane only in a small region, is able to transport a GUV persistently with a speed comparable to that of an isolated self-propelled particle, in agreement with the results on the transport of particle cargo by catalytic Janus micro-motors [35]. If the partial engulfment by the GUV membrane occurs in a large particle region, the Janus particle experiences a membrane curvature-induced force which pushes the Janus particle towards the lowest membrane curvature area, which in turn may quench the particle self-propulsion. 

## Figures and Tables

**Table 1:** Effect of centrifugal force on POPC and DOPC GUVs interacting with MF–Pt Janus colloids, where (●) signifies a significant number of observed events.

MF–Pt Janus Colloids/GUVs	*F_C_* (pN)	Type of Events	
		Adhesion/Engulfment	Membrane Rupture
POPC	10	● (<10%)	
	100	● (30–40%)	
	1000	● (<10%)	●
DOPC	10	● (20–30%)	
	100	● (40–50%)	
	1000	● (<10%)	●

**Table 2:** Effect of centrifugal force on POPC and DOPC GUVs interacting with SiO_2_–Pt Janus colloids, where (●) signifies a significant number of observed events.

SiO_2_–Pt Janus Colloids/GUVs	*F_C_* (pN)	Type of Events	
		Adhesion/Engulfment	Membrane Rupture
POPC	10		
	100	● (<10%)	
	1000	● (<5%)	●
DOPC	10	● (<2%)	●
	100		●
	1000		●

## Data Availability

The data presented in this study are available on request from the corresponding author.

## Abbreviations

The following abbreviations are used in this manuscript:

DOPC	1,2-dioleoyl-sn-glycero-3-phosphocholine
DOTAP	1,2-dioleoyl-3-trimethylammonium-propane
GUV	Giant Unilamellar Vesicle
MF	melamine formaldehyde
POPC	1-palmitoyl-2-oleoyl-glycero-3-phosphocholine
PS	polystyrene

## References

[1] Zhang S., Gao H., Bao G. (2015). Physical Principles of Nanoparticle Cellular Endocytosis. ACS Nano.

[2] Natsume Y., Pravaz O., Yoshida H., Imai M. (2010). Shape deformation of giant vesicles encapsulating charged colloidal particles. Soft Matter.

[3] Agudo-Canalejo J., Lipowsky R. (2015). Critical particle sizes for the engulfment of nanoparticles by membranes and vesicles with bilayer asymmetry. ACS Nano.

[4] Frey F., Idema T. (2021). More than just a barrier: Using physical models to couple membrane shape to cell function. Soft Matter.

[5] Petithory T., Pieuchot L., Josien L., Ponche A., Anselme K., Vonna L. (2021). Size-Dependent Internalization Efficiency of Macrophages from Adsorbed Nanoparticle-Based Monolayers. Nanomaterials.

[6] Dimova R., Marques C.M. (2019). The Giant Vesicle Book.

[7] Deserno M., Bickel T. (2003). Wrapping of a spherical colloid by a fluid membrane. Europhys. Lett..

[8] Dietrich C., Angelova M., Pouligny B. (1997). Adhesion of Latex spheres to giant phospholipid vesicles: Statics and dynamics. J. Phys. II.

[9] Koltover I., Rädler J.O., Safinya C.R. (1999). Membrane mediated attraction and ordered aggregation of colloidal particles bound to giant phospholipid vesicles. Phys. Rev. Lett..

[10] Spanke H.T., Style R.W., François-Martin C., Feofilova M., Eisentraut M., Kress H., Agudo-Canalejo J., Dufresne E.R. (2020). Wrapping of Microparticles by Floppy Lipid Vesicles. Phys. Rev. Lett..

[11] Ewins E.J., Han K., Bharti B., Robinson T., Velev O.D., Dimova R. (2022). Controlled adhesion, membrane pinning and vesicle transport by Janus particles. Chem. Commun..

[12] Vutukuri H.R., Hoore M., Abaurrea-Velasco C., van Buren L., Dutto A., Auth T., Fedosov D.A., Gompper G., Vermant J. (2020). Active particles induce large shape deformations in giant lipid vesicles. Nature.

[13] Sharma V., Azar E., Schroder A.P., Marques C.M., Stocco A. (2021). Active colloids orbiting giant vesicles. Soft Matter.

[14] Love J.C., Gates B.D., Wolfe D.B., Paul K.E., Whitesides G.M. (2002). Fabrication and Wetting Properties of Metallic Half-Shells with Submicron Diameters. Nano Lett..

[15] Weinberger A., Tsai F.C., Koenderink G.H., Schmidt T.F., Itri R., Meier W., Schmatko T., Schröder A., Marques C. (2013). Gel-assisted formation of giant unilamellar vesicles. Biophys. J..

[16] Stocco A., Chollet B., Wang X., Blanc C., Nobili M. (2019). Rotational diffusion of partially wetted colloids at fluid interfaces. J. Colloid Interface Sci..

[17] Shigyou K., Nagai K.H., Hamada T. (2016). Lateral Diffusion of a Submicrometer Particle on a Lipid Bilayer Membrane. Langmuir.

[18] Wang X., In M., Blanc C., Nobili M., Stocco A. (2015). Enhanced active motion of Janus colloids at the water surface. Soft Matter.

[19] Mehr F.N., Grigoriev D., Puretskiy N., Böker A. (2019). Mono-patchy zwitterionic microcolloids as building blocks for pH-controlled self-assembly. Soft Matter.

[20] Klasczyk B., Knecht V., Lipowsky R., Dimova R. (2010). Interactions of alkali metal chlorides with phosphatidylcholine vesicles. Langmuir.

[21] Kollmitzer B., Heftberger P., Rappolt M., Pabst G. (2013). Monolayer spontaneous curvature of raft-forming membrane lipids. Soft Matter.

[22] Goldmans A.J., Cox R.G., Brenner H., Neill O. (1967). Slow viscous motion of a sphere parallel to a plane wall-1 Motion through a quiescent fluid. Chem. Eng. Sci..

[23] Ketzetzi S., De Graaf J., Kraft D.J. (2020). Diffusion-Based Height Analysis Reveals Robust Microswimmer-Wall Separation. Phys. Rev. Lett..

[24] Fragneto G., Charitat T., Daillant J. (2012). Floating lipid bilayers: Models for physics and biology. Eur. Biophys. J..

[25] Cardoso Dos Santos M., Vézy C., Jaffiol R. (2016). Nanoscale characterization of vesicle adhesion by normalized total internal reflection fluorescence microscopy. Biochim. Biophys. Acta Biomembr..

[26] Howse J., Jones R., Ryan A., Gough T., Vafabakhsh R., Golestanian R. (2007). Self-Motile Colloidal Particles: From Directed Propulsion to Random Walk. Phys. Rev. Lett..

[27] Ebbens S., Gregory D.A., Dunderdale G., Howse J.R., Ibrahim Y., Liverpool T.B., Golestanian R. (2014). Electrokinetic effects in catalytic platinum-insulator Janus swimmers. Europhys. Lett..

[28] Brown A., Poon W. (2014). Ionic effects in self-propelled Pt-coated Janus swimmers. Soft Matter.

[29] Spagnolie S.E., Lauga E. (2012). Hydrodynamics of self-propulsion near a boundary: Predictions and accuracy of far-field approximations. J. Fluid Mech..

[30] Agudo-Canalejo J., Lipowsky R. (2017). Uniform and Janus-like nanoparticles in contact with vesicles: Energy landscapes and curvature-induced forces. Soft Matter.

[31] Deserno M. (2004). Elastic deformation of a fluid membrane upon colloid binding. Phys. Rev. E Stat. Nonlinear Soft Matter Phys..

[32] Deserno M. (2004). When do fluid membranes engulf sticky colloids?. J. Phys. Condens. Matter.

[33] Puu G., Gustafson I. (1997). Planar lipid bilayers on solid supports from liposomes—Factors of importance for kinetics and stability. Biochim. Biophys. Acta Biomembr..

[34] Anderson T.H., Min Y., Weirich K.L., Zeng H., Fygenson D., Israelachvili J.N. (2009). Formation of supported bilayers on silica substrates. Langmuir.

[35] Baraban L., Tasinkevych M., Popescu M.N., Sanchez S., Dietrich S., Schmidt O.G. (2012). Transport of cargo by catalytic Janus micro-motors. Soft Matter.

